# Distribution and Abundance of Archaea in South China Sea Sponge *Holoxea* sp. and the Presence of Ammonia-Oxidizing Archaea in Sponge Cells

**DOI:** 10.1155/2011/723696

**Published:** 2011-08-17

**Authors:** Fang Liu, Minqi Han, Fengli Zhang, Baohua Zhang, Zhiyong Li

**Affiliations:** ^1^Marine Biotechnology Laboratory, State Key Laboratory of Microbial Metabolism and School of Life Sciences and Biotechnology, Shanghai Jiao Tong University, 800 Dongchuan Road, Shanghai 200240, China; ^2^Eastern Hepatobiliary Surgery Hospital, Second Military Medical University, Changhai Road 225, Shanghai 200438, China

## Abstract

Compared with bacterial symbionts, little is known about archaea in sponges especially about their spatial distribution and abundance. Understanding the distribution and abundance of ammonia-oxidizing archaea will help greatly in elucidating the potential function of symbionts in nitrogen cycling in sponges. In this study, gene libraries of 16S rRNA gene and ammonia monooxygenase subunit A (*amoA*) genes and quantitative real-time PCR were used to study the spatial distribution and abundance of archaea in the South China Sea sponge *Holoxea* sp. As a result, *Holoxea* sp. specific AOA, mainly group C1a (marine group I: *Crenarchaeota*) were identified. The presence of ammonia-oxidizing crenarchaea was observed for the first time within sponge cells. This study suggested a close relationship between sponge host and its archaeal symbionts as well as the archaeal potential contribution to sponge host in the ammonia-oxidizing process of nitrification.

## 1. Introduction

The biodiversity and biogeography of sponge microbial symbionts has received a great deal of attention, and the past 10 years has witnessed huge advances in revealing the phylogenetic diversity of sponge symbionts. Until the beginning of 2011, 30 bacterial phyla and 2 archaeal phyla have been detected in sponges [1]. However, the role of microbial symbionts remains largely unknown [2–4] and the nature of the sponge-microorganism interaction has to date only been inferred from loose correlations [2]. The present information of sponge microbial symbionts is mainly on the microorganisms in sponge mesohyl, that is, extracellular symbionts [5]. The difficulty in identifying and discriminating between intra- and extracellular symbionts has made it hard to determine the true nature of sponge-microorganism interactions. Therefore, investigation of the intracellular symbionts, which are likely “true” and “stable” symbiotic populations and may play a more significant role in the sponge biology and ecology, is very helpful for the understanding of sponge-microorganism interaction and the roles of sponge microbial symbionts.

Up to now, evidence of intracellular symbionts of sponges is mainly derived from transmission electronic microscopy (TEM) visualization analyses. For example, intracellular algal symbionts in sponges were first confirmed by TEM in 1979 [6]. Using a similar approach, intracellular dinoflagellates [7], filamentous unicellular cyanobacteria [8], and yeast [9] have been observed in sponges. Furthermore, a complex bacterial consortium was revealed in *Ectyoplasia ferox* oocytes using fluorescent in situ hybridization (FISH) in 2008 [10]. Because TEM- or FISH-based methods can provide only limited phylogenetic information, the diversity and abundance of intracellular endosymbionts in sponge cells remain poorly understood.

 Numbers of studies on archaeal sponge symbionts have emerged since 1996 [11–15]. The recent discovery of genes responsible for ammonia oxidation in sponge-associated crenarchaea and evidence of vertical transmission of these symbionts strongly support the argument that these archaea are essential for the metabolism of the sponge host [16, 17]. Though diverse archaea have been observed in sponges [12–15, 18], little is known about the spatial distribution and abundance of archaea in the sponge host and we do not know whether there are archaea in sponge cells. Thus, the examination of the spatial distribution, diversity, and abundance of archaea within sponges especially in sponge cells will greatly help in better understanding the role of archaea play in sponge biology and ecology. 

 In this study, gene library and quantitative real-time quantitative PCR (RT-qPCR) were used to determine the distribution, diversity, and abundance of archaea in the different parts such as cells and mesohyl of South China Sea sponge *Holoxea* sp. The copy number of ammonia-oxidizing genes was also studied to assess the distribution of the AOA community in different parts of sponge *Holoxea* sp. It is the first report of intracellular archaeal symbionts in marine sponges. 

## 2. Materials and Methods

### 2.1. Sampling and Cell Sorting

Marine sponge *Holoxea* sp. was collected nearby Yongxing Island (112°20′E, 16°50′N) in the South China Sea at depth of *ca*. 20 m and processed as described by Li and Liu [19]. Small cubes of sponge tissues (<0.5 cm^3^) were transferred into a 100 mL conical flask and washed using 40 mL sterile artificial seawater (ASW) (1.1 g CaCl_2_, 10.2 g MgCl_2_^.^6H_2_O, 31.6 g NaCl, 0.75 g KCl, 1.0 g Na_2_SO_4_, 2.4 g Tris-HCl, and 0.02 g NaHCO_3_, 1L distilled water, pH 8.2) 3 times for 40 min with shaking at 150 rpm and 20°C. The resulting artificial seawater, which contained extracellular ectosymbionts, was collected, filtered using 300-mesh stainless steel sieve, and further centrifuged at 15,000 ×g to gain extracellular ectosymbionts which refers to microbes loosely attached to the sponge surface and canals, choanocyte chambers (sample W).

**Figure 1:**
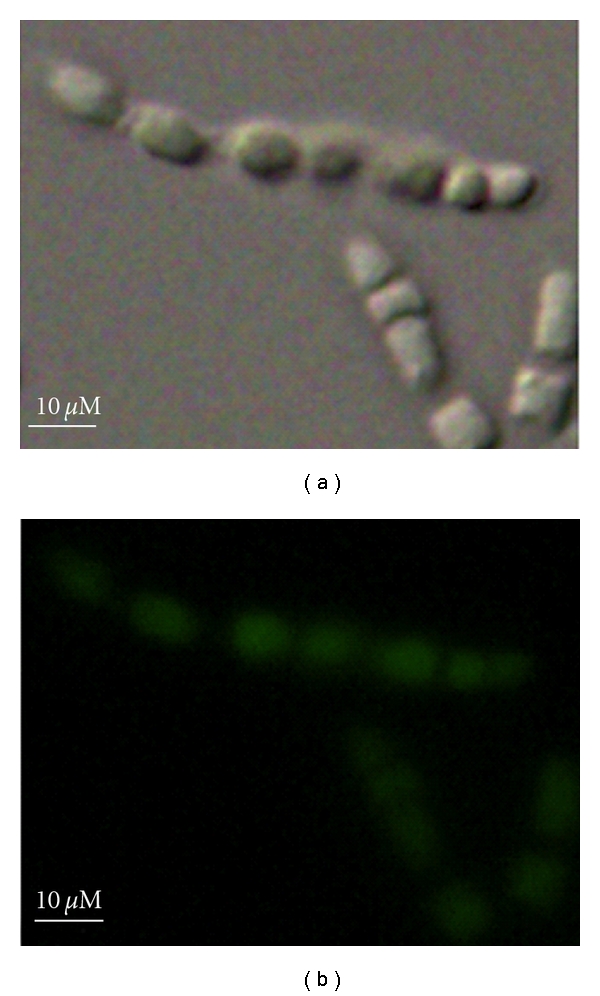
Sponge cells isolated in this study (a) and their autofluorescence (b)  (*λ* = 480 nm).

 The resulting tissue cubes were disintegrated in Ca^2+^- and Mg^2+^-free ASW and were separated using differential centrifugation method described previously [20]. The tissue cubes washed from the previous step were dissociated in Ca^2+^- and Mg^2+^-free ASW at 110 rpm and 20°C for 60 min. The resulting cell suspension was filtered using 300-mesh stainless steel sieve. *Holoxea *sp. has thin outer layer (1-2 mm thick). After 60 min disassociation, outer layer remained intact and was removed through the filtration. Sponge cells, named sample B for analysis of intracellular archaea, were collected by centrifugation at 300 ×g for 10 min, and the supernatant was transferred into a new tube. The resulting pellets were rinsed three times with Ca^2+^- and Mg^2+^-free ASW and identified to be free of bacteria from mesohyl by their autofluorescence (*λ* = 480 nm) (Figure 1). No bacteria-like particulates were found, which proved that the obtained sponge cells were free of bacteria from mesohyl and, thus, were used for diversity analysis of intracellular prokaryotic symbionts of sponge. Supernatants resulted from the previous step were further centrifuged at 15,000 ×g for 10 min. The resulting pellet was named sample J and used to analyze extracellular archaeal endosymbionts (mesohyl).

Sponge tissues without treatments above, named sample T, were used to extract genomic DNA for the analysis of the total communities of bacteria associated with the sponge *Holoxea *sp.

### 2.2. DNA Extraction, Gene Library Construction, and RT-qPCR

Genomic DNA was extracted from samples B, J, and W and sponge specimens (sample T) using the QIAGEN genomic tip protocol. To target the diversity of archaeal community, archaea-specific 16S rRNA gene primer set 21F/958R [21] was used for the construction of 16S rRNA gene libraries, named as BArc, JArc, WArc, and TArc for samples B, J, W, T, respectively. The 16S rRNA gene was amplified using the Arch21F/Arch958R primers with the following PCR condition: 95°C for 3 min; 35 cycles of 95°C for 30 s, 55°C for 30 s, 72°C for 1 min; 72°C for 10 min. Ammonia monooxygenases subunit A (*amo*A) gene was amplified with primer pair Arch-*amo*AF/Arch-*amo*AR [22] from sample T's genomic DNA to construct an *amo*A gene library. The PCR condition: 95°C for 3 min; 35 cycles of 95°C for 30 s, 53°C for 45 s, 72°C for 45 s; 72°C for 5 min. 

The abundance comparison of archaea *amo*A gene between different samples was made using real-time quantitative PCR (SYBR Premix Ex Taq II, Takara) with primer set *amo*A19F/*amo*A643R [23]. As a control, universal archaea 16S rRNA gene primer set 340F/519R [24] was used to quantify the total archaea in the four samples. Specificity for real-time PCR reactions was tested by electrophoresis through a 1.5% agarose gel and melting curve analyses. Copy numbers of *amo*A and 16S rRNA gene were determined using external standards. A standard curve that describes the relationship between archaeal and bacterial *amo*A copy numbers and cycle threshold (CT) values was generated using serial dilutions of a known copy number of the 16S rRNA and *amo*A genes of the plasmid DNA: 16S rRNA, GU227337; *amo*A, GU216235. We calculated the copy numbers directly from the concentration of extracted plasmid DNA by spectrophotometry (Nanodrop Technologies, Rockland, Del, USA). Melting curve analysis was performed from 55°C to 95°C with a reading made every 1°C and the samples held for 1 s between readings.

### 2.3. Statistical and Phylogenetic Analysis

Operational taxonomic units (OTUs) were defined as sequence groups in which sequences differed by ≤1% (2% for *amo*A). Nonparametric richness estimations were performed using DOTUR [25]. A representative clone of each OTU was selected for further phylogenetic analysis. All the OTUs and their closest neighbors determined by BLAST were imported into MEGA 4 [26] for the construction of neighbor-joining trees. Sequences obtained in this study were deposited in the NCBI Genbank under accession numbers: GU227336-GU227339 (16S rRNA archaea) and GU216235-GU216243 (*amo*A archaea).

## 3. Results and Discussion

### 3.1. Distribution and Diversity of Archaeal Symbionts in *Holoxea* sp. 

**Figure 2:**
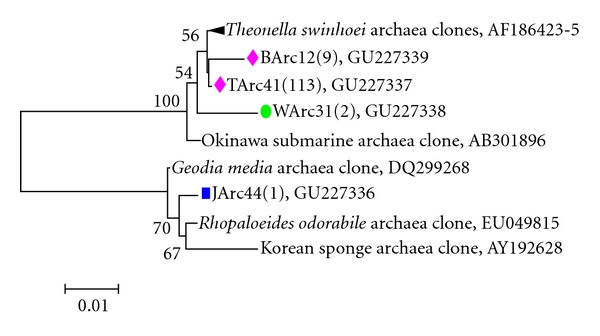
Unrooted 16S rRNA gene-based phylogenetic consensus tree displaying the affiliation of sponge-associated *Crenarchaeota* within group C1a (marine group I: *Crenarchaeota*). Bootstrap values under 50% were cut off after 100 resamplings. Bar: 1 nucleotide substitutions per 100 nucleotides. Numbers in parenthesis stand for the number of clones found in individual library.

According to this study, the archaea community in *Holoxea* sp. was rather simple; all the representative clones in the four groups were identified as group C1a (marine group I: *Crenarchaeota*) and their closest relatives were sponge-derived sequences. Only four OTUs were observed and the biggest one (TArc41) contained 113 clones, including the sequences from all samples. Based on this study, the spatiospecificity for archaea in *Holoxea* sp. was not significant. JArc44 represented the only one singleton (sequence that only occurs in one sample). In phylogenetic tree (Figure 2), these OTUs were divided into two groups: (1) nonsingleton sequences related to *Theonella swinhoei* associated archaea and (2) JArc44 located in another sponge-specific crenarchaeota clade. 

**Figure 3:**
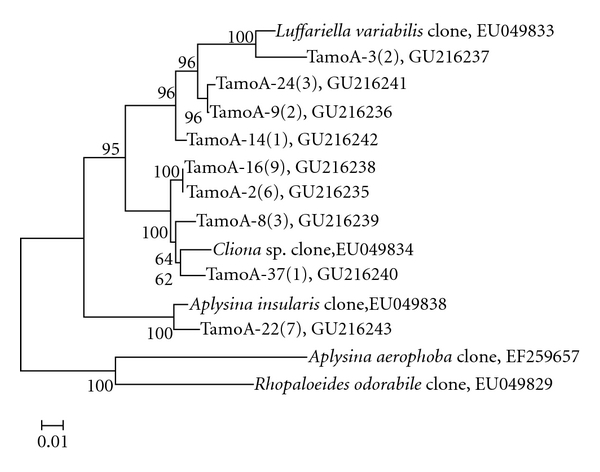
Unrooted *amo*A-based phylogenetic consensus tree of AOA affiliated with the group C1a (marine group I: *Crenarchaeota*). Bootstrap values under 50% were cut off after 100 resamplings. Bar: 1 nucleotide substitutions per 100 nucleotides. Numbers in parenthesis stand for the number of clones found in library.

Analysis of *amo*A gene fragments of sponge sample T revealed a relative high diversity of ammonia-oxidizing archaea (AOA) in sponge* Holoxea* sp. Richness analysis (observed phylotypes/predicted S_ACE_ = 0.8974 and observed phylotypes/predicted S_Chao1_ = 0.9827) indicated that the *amo*A gene library was large enough to yield a stable estimate of phylotype richness. According to the phylogenetic tree in Figure 3, three branches of *Holoxea* sp. associated AOA community including 9 OTUs could be identified based on 2% cutoff. All the *amo*A genes detected were affiliated with the marine group C1a clones [16, 27] and the diversity was noticeable: three branches, respectively, related to *Luffariella variabilis*, *Cliona* sp., and *Aplysina insularis* were identified, which highlighted the ubiquitous distribution of AOA in marine sponges. Almost all the *amo*A genes clustered together suggesting *Holoxea* sp. specific AOA. Comparing to the Figures 2 and 3, the phylogenetic affiliation was not coherent, possibly suggesting that horizontal gene transfer has occurred.

### 3.2. Abundance of AOA Varied in Different Parts of Sponge *Holoxea* sp. 

RT-qPCR displayed an interesting picture, as the proportion of AOA in archaea community indicated in Table 1, the proportion of AOA in intracellular archaeal community (sample J and sample B) was greater than that in extracellular archaeal community (sample W); especially the proportion of intracellular AOA (sample B, 11.67%) was nearly 3-fold that of AOA in sponge mesohyl (sample J, 4.24%), which strongly suggested the presence of AOA within sponge cells. Sponge cells would not uptake microbes randomly [28]. The mechanisms of the presence and transfer of AOA in *Holoxea* sp. are unknown. It has been shown that the microbial community in sponges could be established by vertical transmission [10]. Similarly, sponges may be able to capture AOA by vertical transmission [16]. Archaea of group C1a probably play an important role in the ammonia detoxification within marine sponges [1, 16]. It is known that ammonia oxidation catalyzed by ammonia monooxygenase is the first and rate-limiting step of chemoautotrophic nitrification, the overall oxidation of ammonia to nitrate. Within the sponge body, the AOA would be directly exposed to ammonia released by sponge, so it was suggested that AOA in sponge cells and mesohyl should play a role in ammonia oxidization within the sponge host to remove the toxic ammonia. 

 It was the first time to find *Holoxea* sp. specific AOA, mainly group C1a (marine group I: *Crenarchaeota*), especially intracellular ammonia-oxidizing archaea in sponge cells. Although the detailed function of the plentiful rest archaea in sponge *Holoxea* sp. needs to be investigated, the finding of AOA accumulation in sponge cells in this study indicates the potential role of sponge symbiotic archaea, especially the intracellular AOA in ammonia oxidization, and suggests a close relationship between sponge host and its archaeal symbionts. To further advance our understanding of the diversity and function of intracellular endosymbionts in sponges, metagenomics technology and novel culture methods will be productive approaches.

## Figures and Tables

**Table 1:** Abundance of archaea and AOA in different parts of sponge *Holoxea* sp.

Sample	Copy number^a^		Average proportion of AOA
	*amo*A (AOA)	archaea 16S rRNA	
T	1.71 ± 0.33 × 10^3^	3.36 ± 0.48 × 10^4^	5.10%
W	1.00 ± 0.24 × 10^3^	4.35 ± 0.55 × 10^4^	2.30%
J	2.33 ± 0.09 × 10^3^	5.50 ± 0.31 × 10^4^	4.24%
B	1.89 ± 0.21 × 10^3^	1.62 ± 0.29 × 10^4^	11.67%

^a^Average copy numbers of target gene in one nanogram total genomic DNA. T: whole sponge tissue sample; W: sample of microbes loosely attached to the sponge surface and canals, choanocyte chambers; J: sample of microbes in the sponge mesohyl; B: the intracellular microbes sample.
